# Effects of remimazolam vs. an etomidate–propofol mixture on postoperative cognitive function in elderly female patients undergoing radical mastectomy for breast cancer: a randomized controlled trial

**DOI:** 10.3389/fmed.2025.1699225

**Published:** 2026-01-22

**Authors:** Wenzhe Shen, Lili Li, Ziyi Zhang, Minghong Liu, Jun Shi

**Affiliations:** 1 Graduate School of Bengbu Medical University, Bengbu, Anhui, China; 2 Department of Anesthesiology, The First Affiliated Hospital of Anhui University of Science and Technology, Huainan, Anhui, China

**Keywords:** analgesia, breast cancer, cognitive function, general anesthesia, remimazolam

## Abstract

**Objective:**

This study aimed to evaluate remimazolam’s anesthetic efficacy and impact on postoperative cognitive function in breast cancer patients undergoing radical mastectomy.

**Methods:**

A total of 80 patients were randomized into two groups: Group R (remimazolam, *n* = 40) and Group EP (etomidate–propofol mixture, *n* = 40). Mean arterial pressure (MAP) and heart rate (HR) were recorded at T₀ (pre-induction), T₁ (post-intubation), T₂ (1 h intraoperation), and T₃ (post-extubation). Pain, measured using the visual analog scale (VAS), was assessed upon awakening and at PACU discharge. Cognitive function, measured using the Mini-Mental State Examination/Montreal Cognitive Assessment (MMSE/MoCA), was evaluated on postoperative days 1 and 3. Recovery times and adverse events were also compared between groups.

**Results:**

Baseline characteristics were comparable between groups (*p* > 0.05). At T₁, HR was lower in Group R than in Group EP (*p* < 0.05). At T₂, MAP was higher in Group R (*p* < 0.05). VAS scores showed no intergroup differences postoperatively (*p* > 0.05). MMSE and MoCA scores were significantly higher in Group R at postoperative days 1 and 3 (*p* < 0.05). Following flumazenil antagonism, eye-opening and extubation times were shorter in Group R than in Group EP (*p* < 0.05). The overall adverse event rate was significantly lower in Group R (12.5% vs. 32.5%, *p* < 0.05).

**Conclusion:**

Remimazolam provides effective anesthesia for elderly female patients undergoing radical mastectomy, offering superior hemodynamic stability at key time points, faster recovery, fewer adverse events, and significantly better preservation of early postoperative cognitive function compared with an etomidate–propofol mixture.

**Clinical trial registration number:**

identifier ChiCTR2500106237.

## 1 Introduction

Breast cancer is the most common malignancy and a leading cause of cancer-related death among women worldwide (1). Globally, over one million women are diagnosed with breast cancer annually, with at least 400,000 deaths attributed to the disease, accounting for 14% of all cancer deaths (2–4). Current treatment modalities include endocrine therapy, surgery, radiotherapy, chemotherapy, and biological therapy, with modified radical mastectomy being a primary surgical approach (5). Previous clinical studies have indicated that breast cancer frequently affects middle-aged and elderly women, and the prolonged duration of surgery and anesthesia often contributes to postoperative cognitive dysfunction (POCD) (6). However, in the specific context of radical mastectomy for breast cancer, evaluating postoperative cognitive function carries particular significance that extends beyond routine clinical practice. First, the majority of breast cancer patients are elderly women, who inherently constitute a cognitively vulnerable population. Second, surgery, as a strong physical and psychological stressor, may trigger postoperative cognitive dysfunction (POCD) by inducing neuroinflammation and disrupting neuroendocrine homeostasis (6). More critically, breast cancer patients often need to undergo adjuvant therapies such as chemotherapy and endocrine treatment shortly after surgery, which may further impair cognitive function (7). If POCD develops postoperatively, it could severely compromise patients’ understanding of complex treatment decisions, adherence to long-term therapeutic regimens, and ultimately their quality of life and long-term recovery outcomes. Nevertheless, existing studies on POCD have predominantly focused on orthopedic and abdominal surgeries, while dedicated research targeting radical mastectomy remains relatively limited and often overlooks the cognitive demands posed by postoperative adjuvant treatments. Therefore, implementing neuroprotective anesthesia strategies to reduce the incidence of POCD in elderly patients undergoing radical mastectomy is of considerable clinical importance. Remimazolam, a novel ester-based benzodiazepine sedative, offers advantages such as rapid onset, short duration of action, reversibility (antagonism), minimal accumulation with prolonged infusion, mild cardiovascular depression, and metabolism independent of hepatic or renal function (8). It has been successfully used for procedural sedation and the induction and maintenance of general anesthesia (9, 10). Recent studies have suggested that remimazolam may be superior to midazolam or propofol in preserving postoperative cognitive function following major non-cardiac surgery, lobectomy, and orthopedic surgery in elderly patients (11–13). However, its impact on cognitive function, specifically after radical mastectomy, remains unclear. This study aimed to evaluate the anesthetic efficacy of remimazolam and its effect on postoperative cognitive function in patients undergoing radical mastectomy for breast cancer.

## 2 Materials and methods

### 2.1 General information

This study was approved by the Ethics Committee of the First Affiliated Hospital of Anhui University of Science and Technology (2025-KY-Y029-001), and informed consent was signed by the patients or their family members. From July to September 2025, a total of 102 female patients scheduled to undergo radical mastectomy for breast cancer at the First Affiliated Hospital of Anhui University of Science and Technology were screened. Of these, 5 patients declined to participate, and 17 did not meet the inclusion criteria. Ultimately, 80 eligible patients were enrolled in this study. Using a random number table, participants were divided into two groups: the remimazolam group (Group R, *n* = 40) and the mixture group (Group EP, *n* = 40). Group R received remimazolam besylate 0.3 mg/kg, while Group EP received a 2:3 mixture of etomidate and propofol.

The inclusion criteria were as follows: (1) pathologically confirmed primary breast cancer scheduled for elective radical mastectomy, TNM stage I–II; (2) age 60–75 years; (3) American Society of Anesthesiologists (ASA) physical status II–III; and (4) body mass index (BMI) of 22–28 kg/m^2^.

The exclusion criteria were as follows: (1) Severe cerebrovascular disease, craniocerebral trauma, or other conditions affecting cognitive assessment; (2) history of psychiatric disorders or use of sedatives, hypnotics, or psychotropic drugs within the past 6 months; (3) severe cardiac, pulmonary, hepatic, renal, or metabolic diseases contraindicating general anesthesia; (4) allergy to any study drug component; (5) adrenocortical insufficiency; (6) surgery duration >3.5 h; (7) intraoperative blood transfusion; and (8) intraoperative blood loss >500 mL.

### 2.2 Anesthetic protocol

All patients fasted for 8 h and abstained from clear fluids for 2 h preoperatively. Upon arrival in the operating room, supplemental oxygen was administered, standard monitoring [ECG, SpO₂, and non-invasive blood pressure (NIBP)] was initiated, and peripheral intravenous access was established. Anesthesia was induced intravenously: Group R received remimazolam besylate 0.3 mg/kg, and Group EP received the etomidate–propofol (2:3) mixture 0.2 mL/kg. Upon loss of consciousness, sufentanil (0.5 μg/kg) and rocuronium (0.7 mg/kg) were administered. Tracheal intubation was performed, and mechanical ventilation was initiated with a tidal volume of 6–8 mL/kg, a respiratory rate of 12–14 breaths/min, and FiO₂ of 100%, maintaining end-tidal CO₂ (EtCO₂) between 35 and 45 mmHg. Anesthesia was maintained as follows: Group R received remimazolam besylate infusion at 0.4–1.2 mg/(kg·h), and Group EP received etomidate–propofol mixture infusion at 20–25 mL/h. All patients received concurrent infusions of remifentanil (0.1–0.3 μg/kg/min) and rocuronium (9–12 μg/kg/min). Vital signs and depth of anesthesia (Bispectral Index, BIS) were monitored continuously, maintaining BIS values between 40 and 60. Rocuronium infusion was discontinued 30 min before the anticipated end of surgery, and 10 mg of nalbuphine along with 5 mg of dexamethasone were administered intravenously for preemptive analgesia and antiemesis. Target-controlled infusions of sedatives and analgesics were stopped upon skin closure. Sugammadex (2 mg/kg) was administered at the end of skin closure to reverse residual neuromuscular blockade. Hypotension [defined as systolic blood pressure (SBP) < 20% of baseline for ≥1 min] was treated with intravenous ephedrine (6 mg boluses). Sinus bradycardia (HR < 50 beats/min) was treated with intravenous atropine (0.3 mg boluses). Postoperatively, all patients received patient-controlled intravenous analgesia (PCIA) consisting of sufentanil 0.1 mg + azasetron 30 mg + normal saline 0.9% diluted to a total volume of 100 mL, set at a background infusion rate of 2 mL/h, a bolus dose of 0.5 mL, and a lockout interval of 10 min. Respiratory recovery time and awakening time were recorded. Patients in Group R routinely received flumazenil (0.2 mg) for antagonism. If patients reported severe pain (VAS > 4), flurbiprofen axetil (50 mg) was administered as rescue analgesia. To avoid introducing potential bias that could affect the primary outcome measures, local infiltration anesthesia or nerve block analgesia was not utilized in the study.

### 2.3 Observation parameters

#### 2.3.1 Primary outcomes

*Cognitive function:* Cognitive function was evaluated using the Mini-Mental State Examination (MMSE) (14) and the Montreal Cognitive Assessment (MoCA) (15) 1 day before surgery (preop) and on postoperative days 1 and 3. MoCA-Beijing Version (revised in 2011) was used for all assessments to ensure cultural appropriateness and comparability on postoperative days 1 and 3. MMSE scores range from 0 to 30; scores ≥ 27 indicate normal cognition, and scores < 27 suggest cognitive impairment. MoCA scores range from 0 to 30; scores ≥ 26 indicate normal cognition, and scores < 26 suggest cognitive impairment.

*Vital signs:* Heart rate (HR) and mean arterial pressure (MAP) were recorded at T₀ (10 min pre-induction), T₁ (immediately post-intubation), T₂ (1 h after surgery start), and T₃ (post-extubation).

#### 2.3.2 Secondary outcomes

*Postoperative pain:* Pain intensity was assessed using the visual analog scale (VAS, 0–10) (16) upon awakening, upon PACU discharge, and at 24 h and 72 h postoperatively.

*Recovery profile:* Time to spontaneous respiratory recovery, eye-opening time, and extubation time were recorded.

*Adverse events:* The incidence of intraoperative hypotension, sinus bradycardia, respiratory depression, postoperative nausea and vomiting (PONV), emergence agitation, and the requirement for rescue analgesia were recorded.

### 2.4 Randomization and blinding

The allocation sequence was generated using a random number table method (by an independent statistician), and the allocation plan was sealed in envelopes. During the preoperative visit, grouping was performed by one anesthesiologist (anesthesiologist A), while induction and maintenance of anesthesia were carried out by another anesthesiologist (anesthesiologist B) during the surgery. An observer (a nurse) recorded vital signs and relevant data in real time. Postoperative neurological assessment was conducted by the same neurologist. Both the observer and the neurologist were fully blinded to the group assignments. The personnel responsible for enrollment and the personnel in charge of assigning subjects to the intervention group are unable to access the random allocation sequence.

### 2.5 Statistical analysis

The sample size was estimated based on the primary outcome measure [Mini-Mental State Examination (MMSE) score on postoperative day 1]. Referring to preliminary pilot study data, the mean score was estimated to be 26.2 [standard deviation (SD) = 1.1] for Group 1 and 26.9 (SD = 1.0) for Group 2. With *α* set at = 0.05 (two-tailed) and *β* at 0.20, assuming a detectable difference (*Δ*) of 0.7 and the pooled standard deviation (*σ*) of approximately 1.05, 36 patients per group were required, as calculated using the two-sample *t*-test formula. Considering a 10% dropout rate, the final sample size was expanded to 40 patients per group. Statistical analysis was performed using SPSS Statistics 27.0 (IBM Corporation, United States). Continuous variables were assessed for normality using the Shapiro–Wilk test. Normally distributed data were expressed as mean ± standard deviation (SD) and analyzed using independent samples *t*-tests. Non-normally distributed data were expressed as median (interquartile range, IQR) and analyzed using the Mann–Whitney *U*-test. Categorical data were described as frequencies and percentages and analyzed using the chi-squared test or Fisher’s exact test, as appropriate. A *p*-value of <0.05 was considered statistically significant.

### 2.6 Ethical statement

This study involving human participants was approved by the Ethics Committee of the First Affiliated Hospital of Anhui University of Science and Technology (Approval No. 2025-KY-Y029-001). Written informed consent was obtained from all participants prior to their inclusion in the study, ensuring their understanding and voluntary participation. Furthermore, confidentiality and anonymity of the participants were strictly maintained throughout the research process.

## 3 Results

### 3.1 Baseline characteristics

There were no statistically significant differences between the two groups in age, BMI, ASA classification, operative duration, intraoperative blood loss, or intraoperative fluid administration (all *p* > 0.05), indicating comparability (Table 1).

**Table 1:** Comparison of baseline characteristics.

Variable	EP group (*n* = 40)	R group (*n* = 40)	*t*/*χ*^2^ value	*p*-value
Age [years, (x̄ ± s)]	63.73 ± 3.40	62.98 ± 2.42	*t* = 1.045	0.299
BMI [kg/ $m^{2}$ , ( $\bar{x}$ ±s)]	25.15 ± 2.81	25.17 ± 2.33	*t* = 0.019	0.985
ASA grade II/III (*n*)	39/1	38/2	*χ*^2^ = 0	1
Surgery duration (min)	119.00 ± 19.63	116.40 ± 18.11	*t* = 0.616	0.540
Blood loss (mL)	163.75 ± 61.88	159.63 ± 59.07	*t* = 0.305	0.761
Fluid infusion (mL)	2032.50 ± 432.43	2087.50 ± 449.66	*t* = 0.558	0.579

### 3.2 Intraoperative MAP and HR

**Figure 1:**
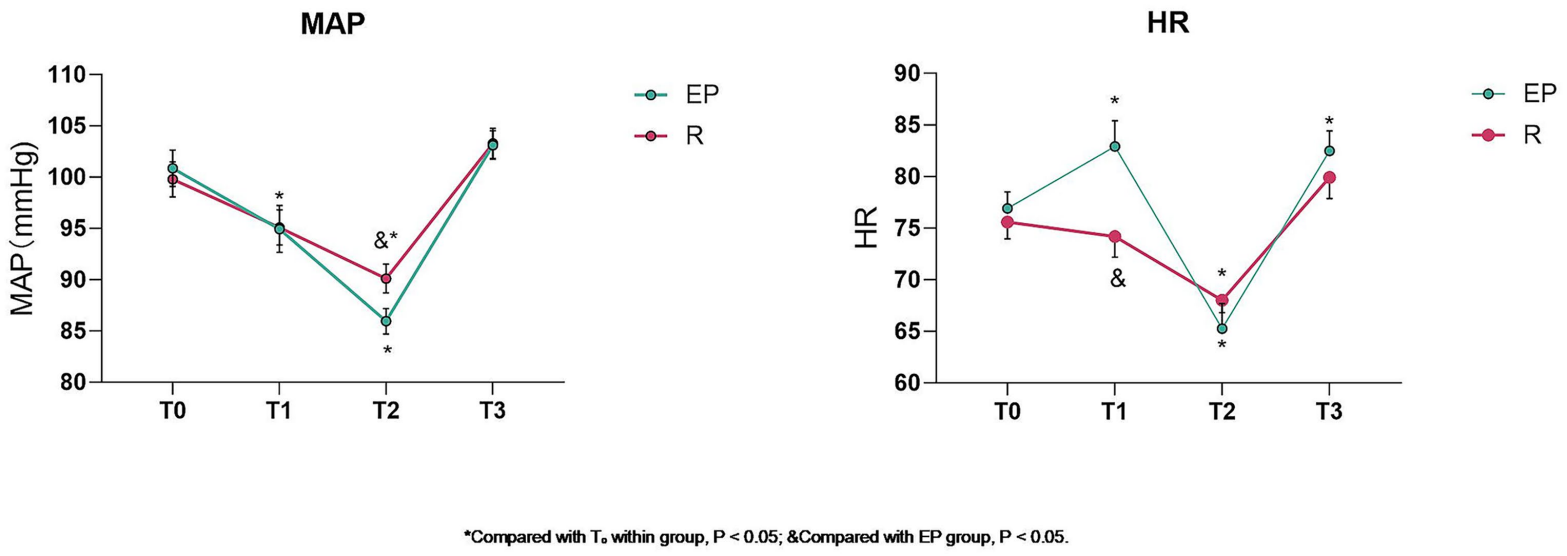
Comparison of MAP and HR between groups (x̄ ± s).* Compared with T₀ within the group, *p* < 0.05; & Compared with the EP group, *p* < 0.05.

No significant differences were observed in MAP between groups at T₀, T₁, T₃ or in HR at T₀, T₂, T₃ (*p* > 0.05). At T₁, HR was significantly lower in Group R compared to Group EP. At T₂, MAP was significantly higher in Group R compared to Group EP (*p* < 0.05). Compared to T₀ within groups: In Group EP, MAP decreased significantly at T₁ and T₂, and HR increased significantly at T₁ and T₃ and decreased significantly at T₂ (*p* < 0.05). In Group R, MAP and HR decreased significantly at T₂ (*p* < 0.05) (Table 2; Figure 1).

**Table 2:** Comparison of MAP and HR between groups (x̄ ± s).

Group	MAP (mmHg)			
	T_0_	T_1_	T_2_	T_3_
EP (*n* = 40)	100.87 ± 11.23	94.94 ± 14.47^*^	85.95 ± 7.85^*^	103.12 ± 8.75
R (*n* = 40)	99.78 ± 10.86	95.10 ± 10.77	90.11 ± 8.83^&*^	103.30 ± 9.32
*t*-value	0.438	0.055	2.226	0.091
*p*-value	0.662	0.956	0.029	0.928

Group	HR (beats/min)			
	T_0_	T_1_	T_2_	T_3_
EP (*n* = 40)	76.90 ± 10.25	82.90 ± 15.96^*^	65.25 ± 15.37^*^	82.48 ± 12.23^*^
R (*n* = 40)	75.60 ± 10.37	74.18 ± 12.57^&^	68.00 ± 7.57^*^	79.90 ± 12.95
*t*-value	0.564	2.144	1.015	0.914
*p*-value	0.574	0.035	0.313	0.363

* Compared with T₀ within group, *p* < 0.05; & Compared with the EP group, *p* < 0.05.

### 3.3 Postoperative pain

No significant differences were observed in VAS scores between the two groups upon awakening, upon PACU discharge, or at 24 h and 72 h postoperatively (*p* > 0.05) (Table 3).

**Table 3:** Comparison of VAS scores between groups [M(Q_1_, Q_3_)].

Group	VAS scores			
	Upon awakening	PACU discharge	Postop 24 h	Postop 72 h
EP (*n* = 40)	2 (1, 3)	2 (2, 3)	2 (2, 1)	1 (0, 1)
R (*n* = 40)	2 (1, 3)	2 (1, 3)	1 (1, 2)	1 (0, 1)
*Z*-value	1.179	1.584	1.598	0.114
*p*-value	0.238	0.113	0.110	0.910

Compared with the EP group, *p* < 0.05.

### 3.4 Cognitive function

**Figure 2:**
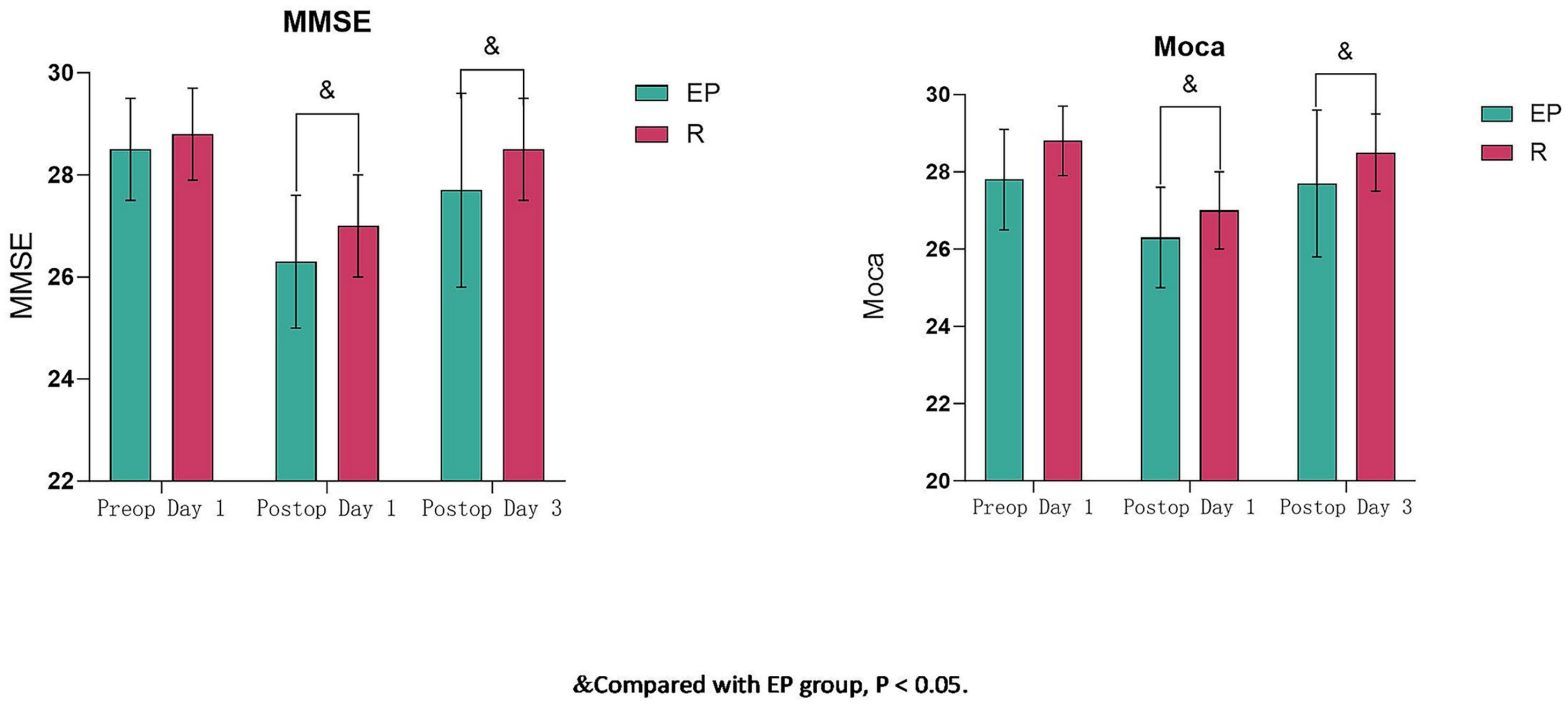
Comparison of MMSE and MoCA scores between groups (x̄ ± s). & Compared with the EP group, *p* < 0.05.

Preoperative MMSE and MoCA scores showed no significant differences between groups (*p* > 0.05). Cognitive function (MMSE and MoCA) decreased significantly on postoperative day 1 compared to preoperative values in both groups (*p* < 0.05). On postoperative days 1 and 3, both MMSE and MoCA scores were significantly higher in Group R compared to Group EP (p < 0.05) (Table 4; Figure 2).

**Table 4:** Comparison of MMSE and MoCA scores between groups (x̄ ± s).

Scale	Time	EP (*n* = 40)	*R* (*n* = 40)	*t*-value	*p*-value
MMSE (score)	Preop Day 1	28.5 ± 1.0	28.8 ± 0.9	1.443	0.153
	Postop Day 1	26.3 ± 1.3	27.0 ± 1.0^&^	2.602	0.011
	Postop Day 3	27.7 ± 1.9	28.5 ± 1.0^&^	2.318	0.023
MoCA (score)	Preop Day 1	27.8 ± 1.3	28.1 ± 1.1	0.949	0.346
	Postop Day 1	25.4 ± 1.6	26.2 ± 1.7^&^	2.139	0.034
	Postop Day 3	27.3 ± 1.2	28.0 ± 1.0^&^	3.130	0.003

& Compared with the EP group, *p* < 0.05.

### 3.5 Recovery quality

Following flumazenil antagonism in Group R, eye-opening time and extubation time were significantly shorter in Group R compared to Group EP (p < 0.05). Respiratory recovery time did not differ significantly between groups (*p* > 0.05) (Table 5).

**Table 5:** Comparison of recovery quality indicators between groups [min, (x̄ ± s)].

Group	Resp. recovery time	Eye-opening time	Extubation time
EP (*n* = 40)	6.31 ± 0.97	10.82 ± 0.81	15.17 ± 1.03
R (*n* = 40)	6.03 ± 0.78	9.91 ± 1.20^&^	14.53 ± 1.10^&^
*t*-value	1.438	3.994	2.700
*p*-value	0.155	<0.01	<0.01

&, Compared with the EP group, *p* < 0.05.

### 3.6 Adverse events

The overall incidence of adverse events was significantly lower in Group R (12.5%) compared to Group EP (32.5%) (*χ*^2^ = 4.588, *p* = 0.032) (Table 6).

**Table 6:** Comparison of adverse events between groups [*n*, (%)].

Group	EP (*n* = 40)	*R* (*n* = 40)	*χ*^2^ value	*p*-value
Respiratory depression	2 (5.0)	0 (0.0)	0.513	0.474
PONV	3 (7.5)	1 (2.5)	0.263	0.608
Emergence agitation	2 (5.0)	1 (2.5)	0.513	0.474
Hypotension	5 (12.5)	1 (2.5)	1.622	0.203
Bradycardia	4 (10)	2 (5)	0.180	0.671
Rescue analgesia	3 (7.5)	2 (5)	0.000	1.000
Total	13 (32.5)	5 (12.5)	4.588	0.032

## 4 Discussion

Radical mastectomy is a primary treatment modality for breast cancer (17). These procedures are typically lengthy and involve significant surgical incisions, and patients are often older women who may experience preoperative anxiety, leading to a relatively high incidence of perioperative complications such as POCD, PONV, and emergence agitation (17). Maintaining hemodynamic stability during surgery, optimizing recovery quality, and minimizing postoperative adverse events are crucial for reducing POCD in this population.

Propofol and etomidate are commonly used anesthetic agents. Propofol combined with analgesics and muscle relaxants is frequently used for a radical mastectomy. Propofol induces sedation by enhancing GABAergic inhibition (18). However, studies suggest that propofol alone provides insufficient anesthesia depth; increasing the dose risks vagal inhibition, potentially causing hypotension and respiratory depression, thereby adversely affecting outcomes (19). While etomidate causes less respiratory depression, it can suppress adrenal function and exert neurotoxicity (5). Clinically, we observed that a mixture of etomidate and propofol offers more stable hemodynamics and fewer adverse events compared to either agent alone. Studies confirm the physicochemical stability of this mixture at room temperature, supporting its clinical use (20). Hence, we utilized a 2:3 etomidate–propofol mixture as the control in this study.

Remimazolam, a novel ultra-short-acting benzodiazepine and GABA_A receptor agonist, provides sedation, hypnosis, and anxiolysis (21). Its mechanism involves enhancing GABA-mediated inhibitory neurotransmission (22). Remimazolam offers a rapid onset, short duration, organ-independent metabolism, minimal respiratory and cardiovascular depression, and promotes hemodynamic stability (23). Our findings revealed that, while MAP showed a decreasing trend post-intubation with remimazolam, it remained comparable to pre-induction levels. Although MAP decreased in both groups at T₂, it was significantly higher in the remimazolam group. HR post-intubation remained stable in Group R but increased significantly in Group EP. Furthermore, Group R had lower incidences of intraoperative hypotension and bradycardia, collectively indicating superior hemodynamic stability with remimazolam.

VAS scores at various postoperative time points showed no significant differences, consistent with most literature. However, Group R required less rescue analgesia, potentially due to sample size limitations. Some studies have suggested that remimazolam may attenuate stress hormone secretion (24), potentially contributing to analgesic effects.

Significantly higher MMSE and MoCA scores at postoperative days 1 and 3 in Group R indicate remimazolam promotes early cognitive recovery. POCD is a common neurological complication, potentially linked to postoperative inflammation and stress hormone release (25). Remimazolam has been shown to suppress pro-inflammatory cytokine release (e.g., IL-1β and TNF-*α* in the hippocampus), reduce postoperative serum levels of norepinephrine (NE), cortisol (Cor), and adrenocorticotropic hormone (ACTH), and mitigate HPA axis hyperactivation induced by surgical stress, thereby protecting neuronal function (26, 27). Our results are consistent with these findings, demonstrating that remimazolam exerts a lesser negative impact on postoperative cognitive function compared to the propofol-based mixture, thereby reducing POCD incidence.

Unlike propofol, remimazolam is specifically antagonized by flumazenil (28). Routine flumazenil administration in Group R, although not significantly altering respiratory recovery time, resulted in significantly shorter eye-opening and extubation times compared to Group EP, indicating superior recovery quality and highlighting remimazolam’s safety profile. Furthermore, remimazolam exhibits no accumulation, and its metabolites lack significant CNS depressant effects, contributing to faster recovery (21). The significantly lower overall adverse event rate in Group R further confirms the safety of remimazolam and its potential to improve postoperative outcomes.

### 4.1 Limitations

This study has several limitations. First, all participants were elderly female patients; therefore, the conclusions apply only to this specific demographic and may not be generalizable to male or younger patients. Second, aligned with the practical workflow of consecutive operating room schedules, flumazenil was routinely administered for reversal only in the remimazolam group. This asymmetric intervention may have potentially influenced recovery parameters between the two groups. Third, this trial was conducted at a single center with a relatively small sample size. Future research should explore the effects of remimazolam on postoperative inflammatory markers and stress hormones and the underlying mechanisms of cognitive protection. Therefore, larger, multi-center studies are warranted.

## 5 Conclusion

Remimazolam can provide effective and satisfactory anesthetic efficacy for elderly female patients undergoing radical mastectomy. Compared to an etomidate–propofol mixture, it offers superior hemodynamic stability during surgery, faster recovery (shorter eye-opening and extubation times), a lower incidence of adverse events, enhanced safety, and better preservation of early postoperative cognitive function.

## Data Availability

The raw data supporting the conclusions of this article will be made available by the authors, without undue reservation.
