# Supplementary material for: Effects of remimazolam vs. an etomidate–propofol mixture on postoperative cognitive function in elderly female patients undergoing radical mastectomy for breast cancer: a randomized controlled trial
Source: Front Med (Lausanne). 2026 Jan 22;12:1699225. doi: 10.3389/fmed.2025.1699225 (PMC12872828; doi:10.3389/fmed.2025.1699225)
Supplement: Supplementary file 1 [file Data_Sheet_1.pdf]

Figure 1: CONSORT 2025 Flow Diagram

Flow diagram of the progress through the phases of a randomised trial of two groups (that is, enrolment, intervention allocation, follow-up, and data analysis)

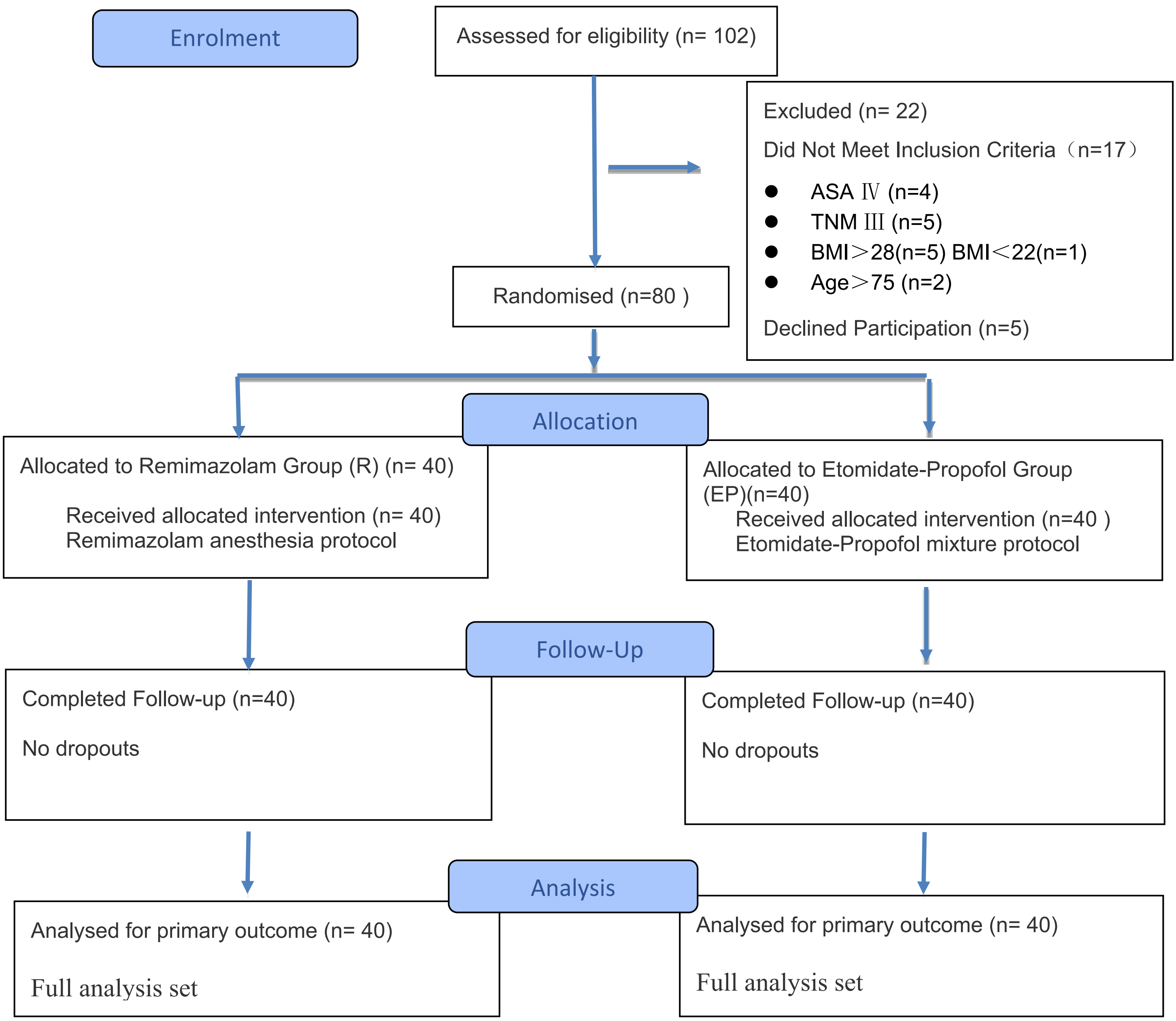

Citation: Hopewell S, Chan AW, Collins GS, Hróbjartsson A, Moher D, Schulz KF, et al. CONSORT 2025 Statement: updated guideline for reporting randomised trials. BMJ. 2025; 388:e081123. https://dx.doi.org/10.1136/bmj-2024-081123
© 2025 Hopewell et al. This is an Open Access article distributed under the terms of the Creative Commons Attribution License (https://creativecommons.org/licenses/by/4.0/), which permits unrestricted use, distribution, and reproduction in any medium, provided the original work is properly cited.
